# Ablation of Calsequestrin-1, Ca^2+^ unbalance, and susceptibility to heat stroke

**DOI:** 10.3389/fphys.2022.1033300

**Published:** 2022-10-12

**Authors:** Feliciano Protasi, Barbara Girolami, Matteo Serano, Laura Pietrangelo, Cecilia Paolini

**Affiliations:** ^1^ Center for Advanced Studies and Technology, University G. d’Annunzio of Chieti-Pescara, Chieti, Italy; ^2^ Department of Medicine and Aging Sciences, University G. d’Annunzio of Chieti-Pescara, Chieti, Italy; ^3^ Department of Neuroscience, Imaging and Clinical Sciences, University G. d’Annunzio of Chieti-Pescara, Chieti, Italy

**Keywords:** Ca^2+^ entry unit (CEU), calsequestrin (Casq), excitation-contraction (EC) coupling, store-operated Ca^2+^ entry (SOCE), malignant hyperthermia susceptibility (MHS), ryanodine receptor (RyR)

## Abstract

**Introduction:** Ca^2+^ levels in adult skeletal muscle fibers are mainly controlled by excitation-contraction (EC) coupling, a mechanism that translates action potentials in release of Ca^2+^ from the sarcoplasmic reticulum (SR) release channels, i.e. the ryanodine receptors type-1 (RyR1). Calsequestrin (Casq) is a protein that binds large amounts of Ca^2+^ in the lumen of the SR terminal cisternae, near sites of Ca^2+^ release. There is general agreement that Casq is not only important for the SR ability to store Ca^2+^, but also for modulating the opening probability of the RyR Ca^2+^ release channels.

**The initial studies:** About 20 years ago we generated a mouse model lacking Casq1 (Casq1-null mice), the isoform predominantly expressed in adult fast twitch skeletal muscle. While the knockout was not lethal as expected, lack of Casq1 caused a striking remodeling of membranes of SR and of transverse tubules (TTs), and mitochondrial damage. Functionally, CASQ1-knockout resulted in reduced SR Ca^2+^ content, smaller Ca^2+^ transients, and severe SR depletion during repetitive stimulation.

**The myopathic phenotype of Casq1-null mice:** After the initial studies, we discovered that Casq1-null mice were prone to sudden death when exposed to halogenated anaesthetics, heat and even strenuous exercise. These syndromes are similar to human malignant hyperthermia susceptibility (MHS) and environmental-exertional heat stroke (HS). We learned that mechanisms underlying these syndromes involved excessive SR Ca^2+^ leak and excessive production of oxidative species: indeed, mortality and mitochondrial damage were significantly prevented by administration of antioxidants and reduction of oxidative stress. Though, how Casq1-null mice could survive without the most important SR Ca^2+^ binding protein was a puzzling issue that was not solved.

**Unravelling the mystery:** The mystery was finally solved in 2020, when we discovered that in Casq1-null mice the SR undergoes adaptations that result in constitutively active store-operated Ca^2+^ entry (SOCE). SOCE is a mechanism that allows skeletal fibers to use external Ca^2+^ when SR stores are depleted. The post-natal compensatory mechanism that allows Casq1-null mice to survive involves the assembly of new SR-TT junctions (named Ca^2+^ entry units) containing Stim1 and Orai1, the two proteins that mediate SOCE.

## The role of Calsequestrin in skeletal muscle function

***Calsequestrin, a member of the macromolecular complex that regulates EC coupling***. Calcium ions (Ca^2+^) are powerful second messengers in all cell types. The electrochemical gradient existing between the cytoplasm and the extracellular space or intracellular stores (either the endoplasmic or sarcoplasmic reticulum; ER and SR) allows release channels to generate elevations in cytoplasmic Ca^2+^ concentrations (transient or prolonged) that may control gene transcription, cellular differentiation, communication between different intracellular organelles, cellular metabolism, *etc.* (Dolmetsch, 2003; Gerke et al., 2005). In muscles, rapid increases in intracellular Ca^2+^ ([Ca^2+^]_i_) regulate contractile function and are generally caused by a large and rapid Ca^2+^ release from internal stores, i.e., sarcoplasmic reticulum (SR), that follows the depolarization of exterior membranes and that may be supplemented, especially in cardiac and smooth muscle, by Ca^2+^ entry from the extracellular space. The mechanism that links depolarization of the sarcolemma to Ca^2+^ release, known as excitation-contraction (EC) coupling (Sandow et al., 1965; Schneider et al., 1973; Rios et al., 1991), is mediated by a coordinated interaction between proteins localized in specialized intracellular junctions named Ca^2+^ release units (CRUs). CRUs are formed by the association of two separate and well-organized membrane systems that come in close contact with one another (Schneider, 1994; Protasi et al., 2002): on one side the sarcolemma and/or the transverse tubule (TT), on the other the internal terminal cisternae of the SR. In skeletal muscle fibers, EC coupling relies on the physical interaction between voltage gated L-type Ca^2+^ channel of TTs, the dihydropyridine receptors (DHPR) also known as CaV1.1, and the SR Ca^2+^ release channel (ryanodine receptor type 1, RyR1). DHPRs and RyRs are the two key elements in the EC coupling machinery (MacLennan and Wong, 1971; Lai et al., 1988). DHPRs are organized in the TT membrane in groups of four, named *tetrads*, which are physically coupled to the 4 identical subunits of RyR1. Several RyR1, which are visible as *feet* in electron microscopy (EM), are clustered in ordered arrays forming usually double rows in the junctional face of SR (Franzini-Armstrong, 1970; Saito et al., 1984; Block et al., 1988; Protasi et al., 1997; Protasi et al., 1998; Protasi et al., 2000; Paolini et al., 2004). DHPRs and RyR1-feet are not the only players in skeletal EC coupling as several other complementary proteins play key roles in EC coupling. Among them, Calsequestrin (Casq) located in the SR lumen of the junctional SR domains (also named terminal cisternae), hence in proximity to RyR1 arrays, plays a key role in providing releasable Ca^2+^ to activate muscle contraction.

***The dual role of Calsequestrin as SR Ca*** ^***2+***^ ***buffer and RyR modulator*** *.* Casq is an acidic protein that binds Ca^2+^ within the SR lumen with high capacity, but medium-low affinity (Meissner, 1975; MacLennan and de Leon, 1983). Casq main function is to provide a large pool of rapidly-releasable Ca^2+^ during EC coupling, mainly acting as a Ca^2+^ buffer in the SR lumen, i.e. a sponge that accumulates ions in proximity of sites of release (MacLennan and Wong, 1971). To play this role Casq is not evenly distributed throughout the SR lumen, but markedly concentrated in correspondence of SR terminal cisternae, in proximity of the junctional face bearing RyR arrays (MacLennan and Wong, 1971; Campbell et al., 1983; Saito et al., 1984). In deep-etch EM studies, Casq appeared as an electron-dense matrix anchored by thin strands, or anchoring filaments, to the SR membrane (Franzini-Armstrong et al., 1987). These strands are likely accessory proteins, i.e. triadin and junctin, that probably allow communication between Casq and RyRs (Kobayashi et al., 2000; Shin et al., 2000; Boncompagni et al., 2012, 2009; Dulhunty et al., 2009; Dulhunty et al., 2009; Wei et al., 2009; Li et al., 2015). In support of this view, Casq over-expression in cardiac muscle resulted in swelling of SR terminal cisternae (Jones et al., 1998; Tijskens et al., 2003), while its knockout caused disappearance of the electron-dense matrix (Knollmann et al., 2006). As active Ca^2+^ transport in the SR lumen is limited by the intraluminal SR free Ca^2+^ concentration (Makinose and Hasselbach, 1965; Weber, 1966; Inesi and de Meis, 1989), Casq presence maintains intraluminal concentrations of free Ca^2+^ low (Beard et al., 2005), allowing for more efficient inward transport by the sarco-endoplasmic reticulum Ca^2+^ ATP-ase (SERCA) pumps. This is especially important in fast-twitch fibers, where the amount of Ca^2+^ cycled in and out of the SR is greater than in slow fibers (Fryer and Stephenson, 1996).

Two isoforms of Casq are expressed in mammalian muscles, products of two different genes: CASQ1 and CASQ2 (Scott et al., 1988; Zarain-Herzberg et al., 1988; Fujii et al., 1990; Arai et al., 1991). Cardiac muscle expresses exclusively Casq2, independently of the developmental stage. On the other hand, in skeletal muscle both isoforms are present: Casq2 is the most abundant isoform in all types of developing skeletal fibers and is still abundantly expressed in adult slow-twitch muscle (Damiani et al., 1990). In fast-twitch fibers, on the other hand, Casq2 is progressively replaced by Casq1, which eventually will remain the only isoform expressed in adult fibers (Sacchetto et al., 1993).

Although evidence from ablation of Casq in *Caenorhabditis (C.) Elegans* wall muscles (which express a single Casq isoform) and in mammalian striated muscles suggested that Casq is not absolutely essential for muscle function (Knollmann et al., 2006; Paolini et al., 2007), there is now agreement that Casq beside being important for the SR ability to store Ca^2+^ (Wang et al., 1998) also modulates the activity of the SR Ca^2+^ release channels, i.e. the RyRs (Ikemoto et al., 1989; Gilchrist et al., 1992; Beard et al., 2002; Beard et al., 2005; Dulhunty et al., 2006). Whereas the specific regulatory function of Casq on the activity of RyR was initially unclear as both activation and inhibition of Ca^2+^ release were reported (Kawasaki and Kasai, 1994; Ohkura et al., 1995; Szegedi et al., 1999; Herzog et al., 2000; Beard et al., 2002; Beard et al., 2005; Dulhunty et al., 2006), there is now general agreement that Casq reduces the opening probability of RyRs.

## Structural and functional consequences of Casq1 ablation in mice

***Ablation of Casq1: the initial findings***. Will the deletion of CASQ1 be lethal, as the knockouts of RYR1 and α_1_DHPR (the 2 key proteins of EC coupling) proved to be (Knudson et al., 1989; Takekura et al., 1995)? Is Casq essential for skeletal muscle function, or the initial findings in *C. Elegans* (Cho et al., 2000) would be also confirmed in mammalian muscle? To address these specific questions, F. Protasi decided in collaboration with his Principal Investigator (Dr. P. D. Allen) and with Lexicon (OmniBank Library, Lexicon Genetics) to start a venture that led to the generation of CASQ1 knockout mice (or Casq1-null). Unexpectedly, Casq1-null mice were viable and fertile, and apparently developed normally under standard housing conditions, raising initially the reasonable doubt that the knockout procedure was unsuccessful. However, western blot (WB) analysis soon confirmed the absence of Casq1 in hindlimb skeletal muscle. In 2002, F. Protasi started the experiments that brough to the publication in 2007 of a first scientific report about Casq1-null mice in *J. Physiol. (London)*. The absence of Casq1 was confirmed by both WB analysis and immunocytochemistry in extensor digitorum longus (EDL) and Soleus muscle, respectively, a predominantly-fast and a predominantly-slow twitch muscle (Paolini et al., 2007). As expected, Casq2 was still present, but its expression was not upregulated to compensate for the lack of Casq1, as initially hypothesized to explain the apparently normal phenotype of knockout mice (Paolini et al., 2007). Casq2 was abundant in Soleus, confined to type I and IIA fibers, but very little Casq2 was found in EDL muscle, mostly composed of fast twitch IIB and IIX fibers. Though, EDL was still functional with most fibers lacking any detectable Casq expression. These initial observations deserved additional investigation focusing on the structural and functional consequences of Casq1 ablation in the EC coupling system.

**FIGURE 1:**
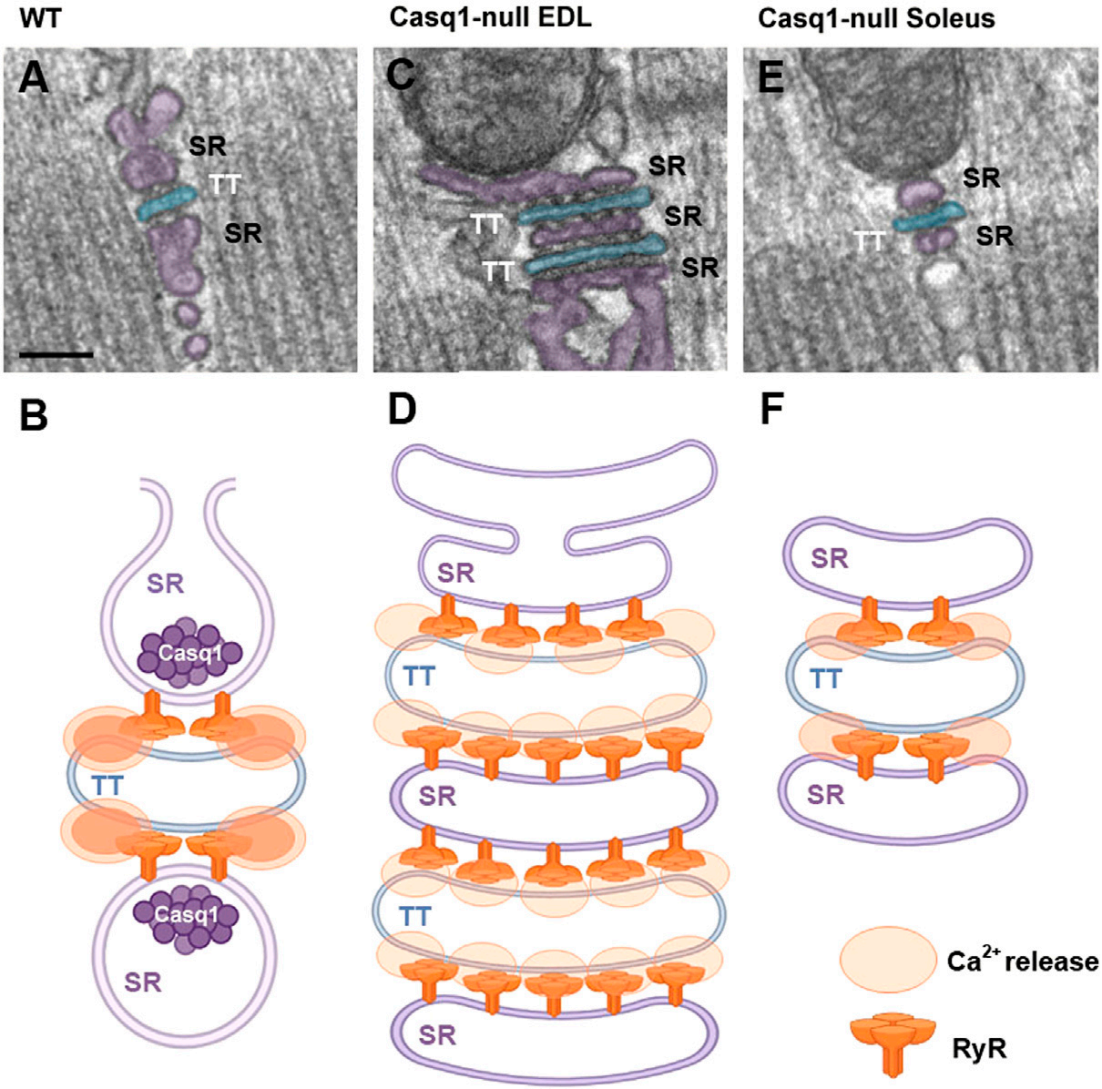
*Representative EM images and schematic cartoons depicting CRUs in WT and Casq1-null fibers.* **(A,B)** In WT fibers, CRUs are formed by three elements (2 SR terminal cisternae flanking a central TT), SR has a wide profile and contains a dark matrix representing Casq. RyRs usually form two rows spanning the junctional gap between SR and TT. **(C–F)** In Casq1-null fibers SR terminal cisternae are significantly narrower than in WT as they lack Casq-matrix; in EDL fibers CRUs are often formed by multiple elements decorated by multiple rows of RyRs. Scale bars: **(A, C, E)**, 0.1 μm.

***The structural adaptations of the EC coupling system to lack of Casq1*** *.* The EM analysis of Casq1-null EDL and Soleus fibers revealed a significant remodeling of the internal membranes involved in EC coupling, i.e. SR and TTs, though different in the two muscles (Figure 1). In Soleus the most evident alteration in EC coupling sites (i.e. CRUs) was a significant reduction in size of SR terminal cisternae, a shrinkage that was also present in EDL fibers (Paolini et al., 2007). In EDL, though, the structural impact of Casq1 ablation was not limited to the reduction in size of the SR lumen in terminal cisternae, but a striking remodeling of CRUs caused the formation of SR-TT-SR junctions often formed by multiple elements (5, 7, even 9) (Paolini et al., 2007), while in control muscle CRUs are usually formed by only three elements (2 SR terminal cisternae flanking a central TT) which are named triads. Furthermore, the Ca^2+^ release channels (RyRs) in these multi-layered CRUs were not organized in two rows as in normal triads but formed by multiple rows similar the arrays of RyR in dyads of cardiomyocytes (Sun et al., 1995; Protasi et al., 1996; Franzini-Armstrong et al., 2005). These findings suggested that multi-layered junctions bearing multiple rows of RyR-feet should contain more RyR1 than normal wild type (WT) fibers, a fact that was promptly confirmed by counting RyR-feet in EM and by ryanodine-binding studies (Paolini et al., 2007).

The shrinkage of terminal cisternae in both Soleus and EDL fibers could be quite easily explained by the lack of Casq1 in the SR lumen of terminal cisternae. Indeed, C. Franzini-Armstrong in a series of elegant papers demonstrated that: a) the SR terminal cisternae is filled with an electron-dense matrix (anchored to the SR membrane by thin strands) and proposed that this matrix was polymerized Casq (Franzini-Armstrong et al., 1987); b) the amount of Casq expressed in muscle fibers can influence size and architecture of the junctional SR (Jones et al., 1998; Tijskens et al., 2003; Knollmann et al., 2006; Franzini-Armstrong, 2009). Hence, in Casq1-null fibers the reduction in size of the SR terminal cisternae is very likely the direct effect of lack of Casq1 in the SR lumen. In support of this hypothesis came studies of re-expression of Casq1 in Casq1-null fibers, which restored the width of junctional SR (Tomasi et al., 2012).

On the other hand, why the membranes of CRUs of EDL fibers proliferate to form multilayered junctions remains to be determined. One hypothesis is that the lack of Casq1 causes a reduction of releasable Ca^2+^ (a fact that was indeed confirmed in functional studies; see below), which in turn would trigger compensatory mechanisms aiming to increase junctional SR volume and/or the number of release sites (i.e. more RyR1 channels available for Ca^2+^ release). Multilayered CRUs do not form in Soleus fibers even when both Casq1 and 2 were missing (Paolini et al., 2011), suggesting that their formation is not the direct result of lack of Casq, but possibly an adaptation of EDL fast-twitch fibers which need more and larger transients than slow-twitch fibers to supply Ca^2+^ ions to the contractile apparatus.

***Other structural modifications caused by Casq1 ablation in skeletal fibers***. While studying the EC coupling machinery (Paolini et al., 2007), we noticed that EDL fibers from Casq1-null mice exhibited areas of partial disarray of the contractile apparatus and swollen/damaged mitochondria. In Paolini et al., 2015, the authors studied more in depth these fiber damage using histological sections, and electron/confocal microscopy. Authors found that Casq1-null mice, beside the remodeling of membranes involved in EC coupling previously described (Paolini et al., 2007), also developed a mild myopathy characterized by mitochondrial damage (starting in adulthood), that eventually caused the formation of structural and contracture cores at later ages (Paolini et al., 2015). Misalignment of contractile elements, Z-line streaming, and regions in which myofibrils were over-contracted progressively increased from 4–6 months to 14–24 months of age (Paolini et al., 2015). Interestingly, similar alterations were described previously in biopsies from patients diagnosed with Central Core Disease (CCD) and in RYR1-knockin mice carrying human mutations associated to CCD (Boncompagni et al., 2009b; Zvaritch et al., 2009). Muscle damage was accompanied by a significant decrease in body weight across all ages (compared to age-matched WT mice), which at least in part could be ascribed to a reduction of muscle mass (Paolini et al., 2015). Cross-sectional area (CSA) of skeletal fibers (Paolini et al., 2015) was on average reduced: one possible mechanism underlying the atrophy of muscle fibers from Casq1-null mice was up-regulation of four atrogenes (CathepsinL, Bnip3, Psmd1, and Atrogin1) that belong to the autophagy and ubiquitin-proteasome pathways, as assessed by microarray analyses of EDL muscles (Paolini et al., 2015). Even though, the molecular analysis found increased expression of PGC-1α, a transcriptional co-activator that stimulates mitochondrial biogenesis and promotes the remodeling of muscle tissue toward a more oxidative metabolism (Lin et al., 2005; Liang and Ward, 2006), histological analyses of EDL muscles from Casq1-null mice did not find fiber-type switching. In turn, the overexpression of PGC-1α could be mediated by the increased cytosolic [Ca^2+^] in Casq1-null fibers (Dainese et al., 2009).

Why ablation of Casq1 leads to mitochondrial damage and formation of cores is still unclear. Though, we know that in fast-twitch fibers of mice most mitochondria are positioned in proximity to CRUs, linked to them by *tethers* (Boncompagni et al., 2009a; Protasi et al., 2021b). It is also well established that mitochondrial function depends on Ca^2+^ release during EC coupling (Rudolf et al., 2004; Rossi et al., 2011) as Ca^2+^ entry into the mitochondrial matrix functions as a signal that activates aerobic ATP production (Jouaville et al., 1999; De Stefani et al., 2011; Gherardi et al., 2020). The proximity between mitochondria and CRUs may be crucial for proper Ca^2+^ signaling between the two organelles (Boncompagni et al., 2009a; Rossi et al., 2009; Rossi et al., 2011). This considered, one could hypothesize that Casq1 ablation causes SR Ca^2+^ leak (due to lack of Casq1 inhibition of RyR1 opening probability) (Beard et al., 2002; Beard et al., 2005), which in turn could: a) increase energy demand for Ca^2+^ re-uptake; and/or b) promote mitochondriogenesis to provide additional energy and/or also cytosolic Ca^2+^ buffering capacity: the volume of mitochondria in Casq1-null fibers was indeed increased (Paolini et al., 2007), possibly as a result of increased expression of PGC-1α; c) cause excessive Ca^2+^ entry that could damage mitochondria: studies conducted in single fibers from double Casq-null mice - which lack both Casq1 and 2 (dCasq-null) - revealed that the mitochondrial [Ca^2+^]_free_ at rest was higher compared to WT (Scorzeto et al., 2013).

The increased structural damage of mitochondria could be the result of a combined action of increased cytosolic Ca^2+^ and also of a redox imbalance. Indeed, the decrease in GSH/GSSG ratio and increase in mSOF activity in muscle from Casq1-null mice were both indicative of increased oxidative stress (Wang et al., 2008; Dainese et al., 2009; Michelucci et al., 2015; Paolini et al., 2015). Mitochondria exposed to excessive cytosolic Ca^2+^ may not function properly and, thus, produce excessive oxidative species of oxygen (ROS), leading to a dangerous feed-forward mechanism that may underline mitochondrial and cellular damage. Membrane structures of the cells can be affected by increased oxidative stress which is known to cause lipid peroxidation (Davies et al., 1982). This vicious cycle resembles that described in mouse model which carries a RYR1^Y522S/WT^ mutation linked to MHS in humans (Durham et al., 2008; Boncompagni et al., 2009b; Wei et al., 2011).

How the mitochondrial damage will later lead to formation of structural cores also remains to be defined. Mitochondrial damage could lead to release of their content, which is known to be a proapoptotic signal (Liu et al., 1996; Zou et al., 1997; Goldstein et al., 2000; Wang, 2001; Garrido et al., 2006; Munoz-Pinedo et al., 2006; Green and Llambi, 2015). In fact, when mitochondria are damaged, cytochrome-c, a protein which in healthy cells is found only in the mitochondrial intermembrane space, is released from the mitochondrial electron transfer chain and initiates caspase activation (Liu et al., 1996; Garrido et al., 2006). In addition, increase in cytosolic Ca^2+^ alone may independently of mitochondria activate the proteolytic machinery, which may break down myofibrils and other intracellular components (Gissel, 2005).

***The functional consequence of lack of Casq1 in the SR***. Despite the striking morphological remodelling of intracellular membrane systems involved in EC coupling (possibly a postnatal attempt of muscle to compensate for the lack of Casq1), Casq1-null fibers were still functionally impaired. Studies in whole muscles showed that Casq1 ablation caused compromised Ca^2+^ release and reuptake by the SR, i.e. prolonged time-to-peak and half-relaxation time. Both parameters were more pronounced in EDL than in Soleus (Paolini et al., 2007), a fact that can be easily explained by the greater dependence of EDL on Casq1, as slow-twitch muscles contain more Casq2. The direct measurement of Ca^2+^ efflux from the SR in isolated single flexor digitorum brevis (FDB) fibers (Paolini et al., 2007) revealed: a) significantly smaller Ca^2+^ transients evoked by electrical stimulation in Casq1-null fibers compared to WT; b) greatly reduced amount of Ca^2+^ stored in the SR, as shown by application of 20 mM caffeine in permeabilized fibers.

A few years later two other papers were published and added additional information toward the full understanding of the mechanisms underlying Ca^2+^ handling in fibers lacking Casq1. Using Ca^2+^dyes of new generation that allowed independent measurements of [Ca^2+^]_free_ in myoplasm and SR lumen:

a) Canato and coll. (2010) demonstrated that CASQ1-knockout results in deep SR depletion under high-frequency stimulation. These findings were explained by the absence of Casq1 in proximity of release sites which would lower the amount of releasable Ca^2+^ during repetitive EC coupling activation. The authors also underlined the fact that in Casq1-null fibers the intermediate step of reuptake, directly dependent on the SR [Ca^2+^]_free_, is the mostly affected, both in terms of amplitude and rate constant (Canato et al., 2010).
b) Royer and coll. (2010), using fibers from dCasq-null mice (lacking both Casq1 and 2), while confirming SR depletion in dCasq-null fibers, added elegant information regarding the rate-velocity of Ca^2+^ efflux from the SR in presence or absence of Casq (Royer et al., 2010). In their description of a new parameter named *evacuality* (E), they stated that: a) E is directly proportional to SR Ca^2+^ permeability and inversely to its Ca^2+^ buffering capacity; and b) E starts low and increases progressively as the SR is depleted in WT fibers, while E starts high and decreases upon SR Ca^2+^ depletion in dCasq-null fibers.

Studies of contraction kinetics in dCasq-null mice were also used to investigate possible differences between fast- and slow-twitch muscles (Paolini et al., 2011). Analysis of grip-strength test in dCasq-null mice (compared to Casq1-null) indicated that muscular function of mice is mainly dependent on the Casq isoform. This is not surprising as most muscle groups of mice are mainly fast-twitch, hence dependant mainly on Casq1. Even in Soleus muscles (a slow twitch muscle containing a considerable amount of Casq2) during high-frequency and prolonged stimulation (2 s pulses), tetanic tension showed only a modest decline, and - contrary to EDL - not a great drop in force.

Re-expression of exogenous Casq1 in skeletal muscle fibers of Casq1-null mice restored almost completely, beside the structure of CRUs (see above), also the functional parameter of Ca^2+^ transient. The corrected (structural and) functional parameters were ascribed only to Casq1 re-introduction, as all other SR proteins that could participate in Ca^2+^ homeostasis did not vary their expression level after re-expression of exogenous Casq1 (Tomasi et al., 2012).

## The discovery of the MHS/HS phenotype of Casq1-null mice

***Increased spontaneous mortality rate in Casq1-null mice***. During the years that brought to the first publication in *J. Physiol.* (Paolini et al., 2007), we noticed a significantly increased rate of spontaneous mortality in Casq1-null mice, particularly after 3 months of age. Initially we did not pay enough attention to this phenomenon, but then realized that most of those deaths were registered in male mice while they were housed in reproductive cages (Dainese et al., 2009). This observation suggested a possible contribution of stress due to mating to mortality rate. Reviewing the existing literature, we discovered that a similar phenotype was reported in animals carrying a defect in EC coupling. Indeed, in porcine stress syndrome (PSS), pigs carrying a mutation in RYR1 causing SR Ca^2+^ leak were prone to sudden death during emotional stress due to transport to market, mating, administration of anesthesia, prolonged exposure to elevated ambient temperature and even during vigorous exercise (Jones et al., 1972; Nelson et al., 1972; Fujii et al., 1991). The PSS is similar to malignant hyperthermia susceptibility (MHS), an abnormal reaction caused in humans by administration of halogenated anaesthetics which affects individuals carrying specific mutations in RYR1 (Denborough and Lovell, 1960; Denborough, et al., 1962; Denborough et al., 1970a; Denborough et al., 1970b; Galli et al., 2006; Wu et al., 2006). Though, MH susceptible individuals may trigger hyperthermic episodes also when exposed to high environmental temperature and/or strenuous exercise: some of these crises and exercise-induced rhabdomyolysis may occur in subjects with a family history of MHS (Wyndham, 1977; Pamukcoglu, 1988; Ryan and Tedeschi, 1997; Tobin et al., 2001; Wappler et al., 2001; Davis et al., 2002; Capacchione et al., 2010; Dlamini et al., 2013). In these cases, hyperthermic reactions are not referred to MHS, but as episodes of exertional-environmental heat stroke (HS).

Based on the above knowledge, we developed the hypothesis that ablation of Casq1 in mice could have caused a pathological phenotype that somehow resembled human MHS or HS. This syndrome would have been responsible of sudden death in male mice during mating. In support of this hypothesis, two additional pieces of information:

1. In cardiac muscle, *gain-of-function* mutations in RYR2 and *loss-of-function* mutations in CASQ2 cause a similar phenotype, i.e. catecholaminergic polymorphic ventricular tachycardia (CPVT), which is caused by excessive SR Ca^2+^ leak (Knollmann et al., 2006).

2. Casq1 was proposed to inhibit the RyR1 opening probability (Beard et al., 2002; Beard et al., 2005), suggesting that Casq1 ablation could in principle cause SR Ca^2+^ leak, as *gain-of-function* mutations in RYR1.

Hence, we decided to test the possibility that Casq1-null mice were susceptible to trigger MH/HS episodes in response to halothane and heat.

***The discovery of Casq1-null mice being susceptible to MH and HS episodes***. In Dainese et al., 2009, we first reported the increased mortality rate of male Casq1-null mice and then tested MH/HS susceptibility exposing mice to halothane (2%, 1 h) and heat (41°C, 30 min). Both protocols were previously used to test the phenotype of RYR1-knockin mice expressing human mutations linked to MH (Chelu et al., 2006; Yang et al., 2006; Durham et al., 2008). During both protocols male Casq1-null mice exhibited a high mortality rate (about 80% of male Casq1-null mice died), while females were only slightly more susceptible than WT (Dainese et al., 2009). MH/HS episodes were characterized by whole body contracture, hyperthermia, and rhabdomyolysis. Importantly, both crises induced by halothane and by heat could be completely prevented by prior intraperitoneal injection of dantrolene (Dainese et al., 2009), the only drug approved for acute treatment of MH in humans (Wedel et al., 1995). Regarding the gender difference in susceptibility, a difference in mortality rate between males and females was also reported in many epidemiologic studies of MH in humans (Strazis and Fox, 1993; Sidman and Gallagher, 1995; Brandom and Larach, 2002; Burkman et al., 2007; Migita et al., 2007). EDL muscles dissected after the exposure to the heat challenge were severely damaged, presenting regions of contractures and overstretched sarcomeres, Z-line misalignment, loss of M-line, and SR swelling (Dainese et al., 2009). As muscle damage suggested rhabdomyolysis (a classic feature of MH and HS crises), we isolated intact EDL muscles and subjected them to an *in-vitro contracture test* (IVCT), the gold standard protocol used to diagnose MH in humans (1984). Muscles of Casq1-null mice exhibited sensitivity to both temperature and caffeine and developed contractures more easily than muscles from WT mice (Dainese et al., 2009). Also, intracellular Ca^2+^ levels were increased at physiological temperatures, both in adult FDB fibers and myotubes of Casq1-null mice (Dainese et al., 2009).

Together, these results suggested that the anesthetic and heat sensitivity of Casq1-null mice closely resemble that of pigs affected by PSS and of mouse models of MH/HS carrying gain-of-function mutations in the RYR gene (Ording et al., 1985; Fujii et al., 1991; Yang et al., 2006; Durham et al., 2008). Furthermore, our results reinforced the correlation between MHS and HS and supported the hypothesis that these two syndromes may share a common pathogenic mechanism (Durham et al., 2008; Dainese et al., 2009; Michelucci et al., 2015; Michelucci et al., 2017b). Recently, we have also shown how oxygen consumption and basal metabolic rate may be used as markers susceptibility of mice to MH and HS (Serano et al., 2022).

***Role of oxidative stress in SR Ca^2+^ leak and in hyperthermic crises***. In 2008, it was proposed that a key molecular event leading to hyperthermic crises is the excessive production of oxidative species of oxygen and nitrogen (ROS and NOS) by mitochondria, which in turn would exacerbate SR Ca^2+^ leak *via* RyR1 nitrosylation (Durham et al., 2008). In Michelucci et al., 2015 the efficacy of two different antioxidant compounds in preventing hyperthermic reactions was tested *in-vivo* in Casq1-null mice. N-acetylcysteine (NAC) and Trolox were respectively administrated in drinking water or by intraperitoneal injection. Both treatments were quite successful in reducing the mortality rate of Casq1-null mice in response to exposure to heat (41°C, 1 h) and halothane (2%, 1 h at 32°C): from about ∼80% to only ∼20%. Animals treated with antioxidant also showed a decreased rise in core temperature during the heat challenge and a delayed onset of lethal crises (Michelucci et al., 2015). In addition, NAC treatment also: a) reduced muscle damage during exposure to heat stress (assessed by quantifying: the number of damaged fibers in histology, serum CK levels, and plasma K^+^ and Ca^2+^ concentrations); b) rescued structural alterations observed in Casq1-null mice at rest, reducing mitochondrial damage (Paolini et al., 2015); and finally c) normalized IVCT, lowering the caffeine sensitivity of resting tension in EDL muscles from Casq1-null muscles.

To confirm that antioxidants reduced oxidative stress, authors first measured mitochondrial superoxide flashes (mSOF) activity after the treatment with NAC in dissociated FDB muscle fibers (Wei et al., 2011; Michelucci et al., 2015; Cheng et al., 2018): mSOF frequency (elevated in knockout fibers) was significantly reduced by NAC both at 20° and 37°C (Michelucci et al., 2015). In addition, biochemical analyses performed in EDL muscles revealed that NAC treatment reduced the expression levels of superoxide dismutase-1 (SOD-1), 3-Nitro-l-Tyrosine (3-NT), and finally lowered reduced/oxidized glutathione ratio (GSH/GSSG) in comparison to muscle of untreated Casq1-null mice. As SOD-1 is one of the main antioxidant enzymes (McCord and Fridovich, 1969), 3-NT are direct products of oxidative damage on proteins (Ogino and Wang, 2007), and GSH/GSSG ratio reflect the overall cellular redox state, data collected indicated that NAC treatment reduced oxidative stress (Michelucci et al., 2015).

To verify if the mechanisms underlying the effect of antioxidants involved modulation of intracellular Ca^2+^ levels, the effect of NAC treatment on Ca^2+^ transients was also tested directly in isolated FDB fibers loaded with a ratiometric Ca^2+^ dye in experiments at rest and during prolonged high-frequency stimulation (60 Hz, 2 s) (Michelucci et al., 2015).

In two later studies published between 2017 and 2018, additional evidence pointing to a central role of oxidative stress in HS crises was collected (Michelucci et al., 2017a; Guarnier et al., 2018).

(a) In the first study to investigate the gender difference in the phenotype of Casq1-null mice (female mice being more protected than males during exposure to halothane or environmental heat), we changed the hormonal profile of mice (Michelucci et al., 2017a). Many studies in literature reported a higher percentage of MHS cases in males than females (about 70/80% vs females) (Strazis and Fox, 1993; Larach et al., 2001; Burkman et al., 2007; Migita et al., 2007). The potent cellular antioxidant properties of estrogens, female sex steroid hormones, are well documented in literature: (i) upregulation of several antioxidant enzymes, (ii) downregulation of ROS-generating enzymes, and (iii) direct free-radical scavenging properties (Sugioka et al., 1987; Mooradian, 1993; Gomez-Zubeldia et al., 2001; Wagner et al., 2001; Florian et al., 2004; Borras et al., 2005). To test if estrogens were protecting female mice from MH/HS crises, we treated for 1 month (3–4 months of age): (i) male Casq1-null mice with Premarin (a dose of 40 ng/g of body weight every day for 1 month; McCullough et al., 2001; Shen et al., 2008) and (ii) female Casq1-null mice with Leuprolide (a dose of 100 ng/g of body weight every day for 1 month), known to abolish estrogens production (Wilson et al., 2007).
(b) In the second paper published in 2018, as aerobic training is known to increase endogenous antioxidant protection (Venditti and Di Meo, 1997; de Sousa et al., 2017), we subjected Casq1-null mice to aerobic training on treadmill for 2 months at 60% of their maximal speed (1 h, 5 times/week).

In short, both estrogen administration (Michelucci et al., 2017a) and aerobic training (Guarnier et al., 2018) protected male Casq1-null mice from lethal episodes by reducing hyperthermia during exposure of mice to heat stress. The protective effect of these treatments was also visible in IVCT: the generation of tension of EDL muscles isolated from Casq1-null mice to increasing [caffeine] and/or temperature was reduced by administration of Premarin and also by aerobic exercise. Also, rhabdomyolysis of skeletal muscle fibers and mitochondrial damage were prevented/reduced by estrogens and training in EDL muscle from Casq1-null mice. Analysis of oxidative stress levels confirmed the antioxidant role of estrogens and of aerobic training in muscles of Casq1-null mice: (i) GSH/GSSG ratio, (ii) levels of nitrated proteins (3-NT) (Michelucci et al., 2017a), (iii) levels of carbonyl proteins and (of diene conjugates (Guarnier et al., 2018) were all normalized. Note that administration of Leuprolide to female Casq1-null mice, a treatment that reduces the production of estrogens (Michelucci et al., 2017a), increased the susceptibility of female mice to heat stress. Together, the results contained in these two studies (Michelucci et al., 2017a; Guarnier et al., 2018) reinforced: a) the hypothesis proposed by Durham and coll. (2008) that oxidative stress plays a key role in hyperthermic crises; b) that antioxidants could be in principle used as preventive measure in periods of intense environmental heat to reduce the risk of HS.

***Exertional stress is also a factor of risk for hyperthermic crises in Casq1-null mice***. Humans experience hyperthermic crises with very similar symptoms, either as a result of the administration of volatile anesthetics (malignant hyperthermia, MH), or when exposed to environmental heat or to intense physical/emotional stress (environmental/exertional HS) (Wyndham, 1977; Pamukcoglu, 1988; Ryan and Tedeschi, 1997; Tobin et al., 2001; Wappler et al., 2001; Davis et al., 2002; Capacchione et al., 2010; Dlamini et al., 2013). Some examples: a) muscle biopsies from two soldiers who have experienced episodes of exertional HS were positive to halothane during IVCT (Hopkins et al., 1991); b) MH patients were associated with either non-specific myopathies or congenital disorders in three separate families; two of these patients also showed evidence of exercise-induced rhabdomyolysis (Davis et al., 2002); c) life-threatening episodes triggered by either environmental heat or physical exertion are characterized by hyperthermia and rhabdomyolysis, a pathophysiologic fingerprint also typical of anesthetic-induced MH crises (Hopkins et al., 1991; Bouchama and Knochel, 2002; Davis et al., 2002; Capacchione et al., 2010; Lehmann-Horn et al., 2011).

While susceptibility to anesthetics and environmental heat was tested between 2006 and 2009 in knock-in mice carrying RYR1 mutations linked to MH in humans (Chelu et al., 2006; Yang et al., 2006; Durham et al., 2008) and in Casq1-null mice (Dainese et al., 2009), the susceptibility to physical exertion remained to be tested. For this reason, we subjected RYR1^Y522S/WT^ and Casq1-null mice to an exertional-stress (ES) protocol, i.e. incremental running on a treadmill placed in an environmental chamber in which temperature and humidity were kept at 34°C and 40% respectively (Michelucci et al., 2017c). We also tested the efficacy of drugs commonly used in humans to block MH reactions triggered by administration of anesthetics. The main findings of this study were:

(a) Lethal crises occurred in about 80% of both RYR1^Y522S/WT^ and Casq1-null mice exposed to the ES protocols, while no lethal episodes were registered in WT mice. Of note, the lethal crises during the ES protocol displayed clinical signs very similar to those experienced by the same animals when exposed to either halothane or environmental heat (i.e., whole-body contractures, difficulty in breathing, and impaired and spasmodic movements).
(b) Pretreatment of susceptible mice 60min prior the ES protocol with a single injection of Azumolene (a Dantrolene analog which is more water soluble) was effective in preventing exertional HS: the mortality rate was indeed almost completely abolished in both mouse models, and also core temperature was reduced in pre-treated animals.
c) Azumolene reduced also muscle damage during ES protocol, and normalized IVCT and intracellular Ca^2+^ levels during exposure to increasing [caffeine].
(d) Finally, as in MH crisis triggered by either exposure to halothane and heat ROS and RNS had a pivotal role in promoting a feed-forward mechanism that led to excessive SR Ca^2+^ release (Durham et al., 2008; Michelucci et al., 2015), we measured the levels of 3-NT, a product of nitration of tyrosine residues in proteins mediated by RNS (Ogino and Wang, 2007). Muscles of RYR1^Y522S/WT^ and Casq1-null mice showed higher levels of 3-NT already in basal conditions (compared to WT), and even more following ES; though, pretreatment with Azumolene was effective in controlling the increase in 3-NT levels during exposure to the ES protocol.

The data collected in this study (Michelucci et al., 2017c) suggested: a) MH susceptible individuals could, in principle, be considered at risk for exertion-induced episodes; and b) drugs that are commonly used to treat classic anesthetic MH reactions (i.e., Dantrolene) could be considered as possible pharmacologic interventions for acute HS, a class of disorders that currently has no cure.

## Why Casq1-null mice were viable? Finally solving the mystery

***The role of Store Operated Ca^2+^ entry in skeletal muscle fibers***. Ca^2+^ homeostasis in skeletal fibers is the result of a complex interplay between several mechanisms: release of Ca^2+^ by EC coupling, Ca^2+^ re-uptake by the SR, Ca^2+^ binding to sarcomeric and cytoplasmic proteins (Troponin, Parvalbumin, *etc.*), mitochondrial Ca^2+^ uptake, *etc.* (Calderon et al., 2014) In addition, during prolonged and repetitive stimulation, fibers may lose some Ca^2+^ in the extracellular space *via* plasma membrane Ca^2+^ ATP-ases (PMCA). In turn, this may result in a reduction of the total amount of Ca^2+^ stored in the SR, which is one the main mechanism underlying muscle fatigue (Lamb, 2002). Though, muscle fibers have developed systems to recover extracellular Ca^2+^ when the amount of Ca^2+^ in intracellular SR stores is too low to support normal muscle function: this condition is known as *SR depletion*.

Depletion of intracellular Ca^2+^ stores (ER and SR) in excitable and non-excitable cells is known to be the trigger for the activation of store-operated Ca^2+^ entry (SOCE). SOCE is a ubiquitous Ca^2+^ influx mechanism in which Stim1 (stromal interaction molecule-1), a Ca^2+^ sensor in the lumen of the ER/SR (Liou et al., 2005; Roos et al., 2005; Zhang et al., 2005), and Orai1, a Ca^2+^ release-activated Ca^2+^ (CRAC) channel of external membranes (Feske et al., 2006; Prakriya et al., 2006; Vig et al., 2006; Yeromin et al., 2006), are the two main players. Activation of SOCE is triggered by dimerization of Stim1 in the ER/SR when [Ca^2+^] is too low, which then activates Orai1 in the plasma membrane that allows Ca^2+^entry (Michelucci et al., 2018; Qiu and Lewis, 2019). In muscle, it has been demonstrated that SOCE is also modulated by Casq1: Wang and coll (Wang et al., 2015) reported a retrograde regulation of Stim1-Orai1 interaction (and SOCE) by Casq. Casq seems to exert its role in SOCE through the formation of a complex between the aspartate-rich segment of Casq1 and Stim1, which inhibits Stim1 aggregation (Zhang et al., 2016). As the physiological role of Casq1 would be to inhibit the SOCE mechanism, the deletion of CASQ should in principle upregulate SOCE. Indeed, accelerated activation of SOCE current in myotubes from Casq1-null mice was already reported (Yarotskyy et al., 2013).

**FIGURE 2:**
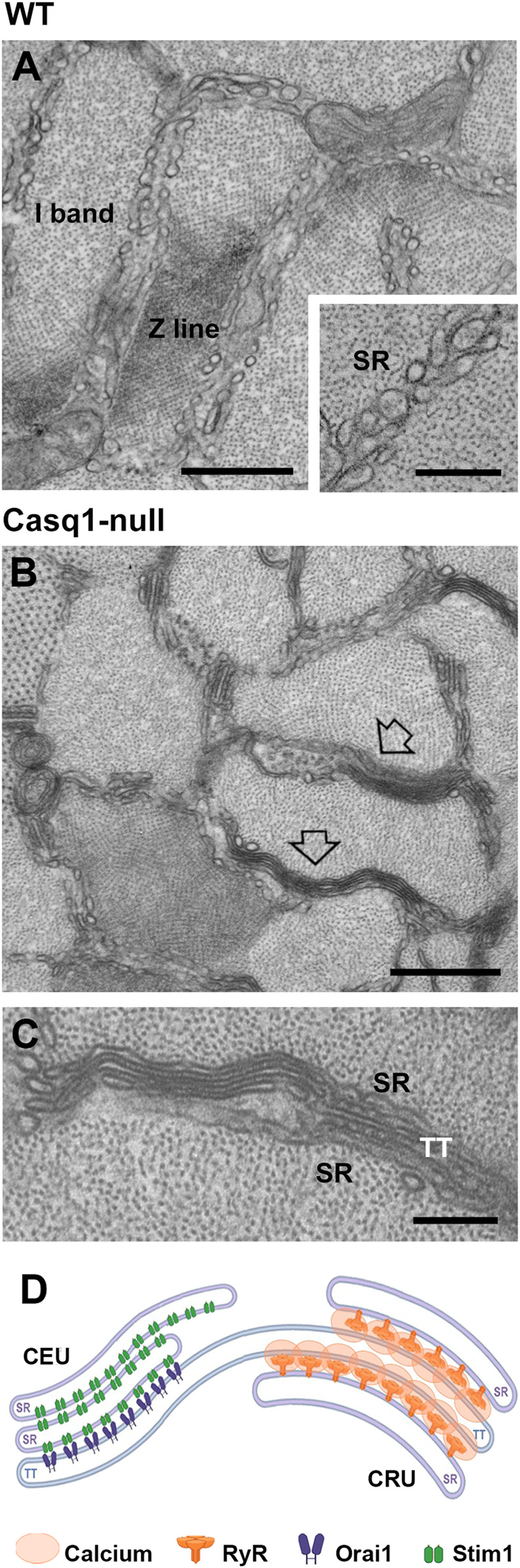
*Representative EM images of SR at the I band of WT and Casq1-null fibers and schematic cartoon of SR remodeling in Casq1-null fibers.* **(A)** SR at I band of WT EDL fibers appear as SR vesicles and small tubes. **(B,C)** In Casq1-null EDL fibers, SR at the I band is often remodeled in SR-stacks (pointed by empty arrows), one the two components of CEUs. **(D)** Cartoon showing a CEU and a CRU, which are both junctions between SR at TT, though they contain different proteins (see symbol legend). Scale bars: **(A,B)**, 0.2 μm; inset in **(A,C)** 0.5 μm.

SOCE activity in muscle is important to limit fatigue during sustained and repetitive activity (Wei-Lapierre et al., 2013; Carrell et al., 2016; Michelucci et al., 2018). As SOCE activation in muscle is apparently faster than in non-excitable cells (Launikonis et al., 2003; Launikonis and Rios, 2007), it was initially proposed that the interaction of Stim1 and Orai1 would occur in CRUs, the sites of EC coupling where TTs and SR (containing respectively Orai1 and Stim1) are already in contact with one another (Dirksen, 2009). However, later work by our group demonstrated that SOCE in muscle occurs in different junctions that are formed at the I band between stacks of SR membranes and extensions of TT, named Ca^2+^ Entry Units (CEUs) (Boncompagni et al., 2017; 2018; Protasi et al., 2021a). These junctions are few and small in muscle at rest, but become larger and more numerous during acute exercise (Figure 2). We demonstrated that CEUs contain Stim1 and Orai1 proteins, which increase their colocalization during exercise thank to elongation of TTs at the I band and remodeling of SR membranes in flat cisternae.

Assembly of CEUs during exercise have been correlated with: a) increased fatigue resistance in presence of external Ca^2+^, which is lost when extracellular Ca^2+^ is removed or Ca^2+^ entry blocked by supplementation in the extracellular solution with SOCE blockers (BTP-2 and 2-APB) (Boncompagni et al., 2017); b) reduced decline of Ca^2+^ transient amplitude during repetitive high-frequency stimulation; c) accelerated rate of Mn^2+^ quench, the gold standard technique used to assess SOCE function (Michelucci et al., 2019). We also reported that CEUs are dynamic structures as they promptly assemble during exercise, but also disassemble in the first hours during recovery, when TTs retract from SR-stacks preventing Orai1 from interacting with Stim1, hence inactivating the function of SOCE (Michelucci et al., 2019).

***Casq1-null fibers contain constitutively assembled Ca^2+^ Entry Units***. As Casq was considered one of the three key proteins on EC coupling (together with RyR1 and DHPR), when we planned to generate Casq1-null mice we expected a lethal phenotype. We were not expecting that muscle fibers could function in absence of Casq1, yet surprisingly mice were alive and developed quite normally to adulthood (see paragraph *Structural and functional consequences of Casq1 ablation in mice*). As detailed above, EM allowed us to discover that lack of Casq1 induced morphological adaptations to sites of EC coupling: CRUs were formed by multiple SR-TT-SR junctions and fibers contained an almost doubled amount of RyR1 (Paolini et al., 2007) (Figure 1). At the time we thought that these structural adaptations must have been sufficient to allow an almost normal function of skeletal fibers. Though, we did not have the foresight to see what we recently discovered (Michelucci et al., 2020).

Before our first publication in 2007, while performing EM to analyse the EC coupling machinery, we did not notice that lack of Casq1 induced also the formation of stacks of flat SR cisternae oriented longitudinally between myofibrils, mostly located at the I band (Figure 2). SR-stacks in Casq1-null fibers were first reported by Boncompagni et al., 2012. Structures virtually identical to these SR-stacks were reported in mice lacking junctophilin, and described as *irregular triad junctions* by Ko and coll. (Ko et al., 2011). After that, SR-stacks were also found in triadin/junctin knockout fibers (Boncompagni et al., 2012), in Casq2-null skeletal fibers (Valle et al., 2016), and in MAP6 knockout muscle (Sebastien et al., 2018).

Almost a decade later the initial observation of stacks in Casq1-null fibers (Boncompagni et al., 2012), and after discovering the sites of SOCE (Boncompagni et al., 2017; 2018; Michelucci et al., 2019), we finally realized that the stacks of flat SR cisternae found in Casq1-null fibers were one of the two main elements forming CEUs, the intracellular junctions that mediate SOCE. The data collected over several years in two separate projects (1. characterization of Casq1-null mice phenotype; 2. identification of SOCE-sites) led us to hypothesize that SOCE activation, through the assembly of CEUs, could be another mechanism used by Casq1-null mice to compensate for the lack of Casq1 in the SR.

Compelling evidence published in Michelucci et al., 2020 in *J. Gen. Physiol.* revealed that EDL muscles from sedentary Casq1-null mice (e.g. not subjected to exercise) contained preassembled junctions between SR-stacks and TT extensions within the I band virtually identical to the CEUs formed in WT mice during acute exercise (Boncompagni et al., 2017; 2018; Michelucci et al., 2019). Quantitative analysis by EM showed that CEUs in Casq1-null fibers were fully assembled as both components were in place: i) higher percentage of fibers with SR-stacks and more SR-stacks per unit area, compared to control muscle; and ii) increased TT extension at the I band and more contacts with SR-stacks, compared to WT muscle. Pre-assembly of CEUs in muscle of Casq1-null mice is likely mediated by increased expression of Stim1S, Stim1L, Orai1, and SERCA. The final proofs that these pre-assembled CEUs were functional came from studies showing: a) increased rate of Mn^2+^ quench in FDB fibers of Casq1-null compared to WT, both following a protocol of SR depletion and without SR depletion; b) increased SR refilling during repetitive stimulation, which helped to normalize Ca^2+^ transients (abolished by removal of external Ca^2+^ or when Ca^2+^ entry was reduced by presence of SOCE inhibitors, e.g. 0 Ca^2+^ or BTP-2).

The findings reported by Michelucci and others demonstrated that the SR-TT junctions within the I band of Casq1-null mice represent pre-assembled CEUs that provide an active pathway for constitutive and store-operated Ca^2+^ influx (Michelucci et al., 2020). Constitutively active SOCE potentially serves as a surrogate Ca^2+^ pool and compensates for the marked reduction in total SR Ca^2+^ store capacity caused by the lack of Casq1 (Paolini et al., 2007; Canato et al., 2010). Pre-assembled CEUs may result from a long-term post-natal adaptation which helps to maintain proper muscle function during prolonged periods of activity.

## Final remarks

We had known for several years that the total amount of releasable Ca^2+^ from intracellular SR stores was markedly reduced in Casq1-null fibers (Paolini et al., 2007). Though, the resistance to fatigue of fibers and mice was not significantly impaired. The increased mitochondrial number/volume (Paolini et al., 2007), which in turn was possibly induced by the need of additional ATP to remove excessive accumulation of Ca^2+^ in the cytoplasm, could have been one factor that helped Casq1-null muscles to sustain prolonged activity. Yet our hypothesis was not correct.

Casq1-null fibers were using external Ca^2+^ to compensate for the low amount of Ca^2+^ stored in the SR. Now also the severe SR depletion of Casq1-null fibers when subjected to high frequency stimulation reported in Canato et al., 2010 makes more sense, as SR depletion is the key signal that activates SOCE, hence assembly of CEUs. In the end, the presence of constitutively active CEUs in muscle of Casq1-null mice could alone explain how these mice are able to survive despite the absence of a protein once considered fundamental for EC coupling and for skeletal muscle function.

## Conclusion

Generation of knockout mice was once a real challenge. However, year-by-year it has become easier and easier to generate knockout and knockin mice to study the function of specific proteins. Often so easy that - my personal feeling - animal models generated with modern and fast techniques, maybe at a lower cost, are often not really investigated in depth. It is more appealing to most scientists to generate a new model, than deepen the investigation on a model that has been already published once or twice.

Our experience with Casq1-null mice has been a long journey of almost 20 years now, that demonstrated to us how the structural and functional changes induced by the ablation of a single protein may be quite complex, and definitely not easy to unravel with a single - not even a few - publications. First, we described morphological and functional changes induced by ablation of Casq1 in adult mice: remodeling of CRUs with more Ca^2+^ release channels and reduced SR Ca^2+^ content, which caused fast depletion under high frequency stimulation (Paolini et al., 2007; Canato et al., 2010). Then, spontaneous mortality of male mice pushed us to investigate these deaths and allowed the discovery of susceptibility of mice to trigger hyperthermic episodes when exposed to halothane, heat, and even strenuous exercise (Dainese et al., 2009; Protasi et al., 2009; Michelucci et al., 2017c). During these investigations we were also able to deepen our understanding of the molecular bases of these disorders (Ca^2+^ leak, oxidative stress; mitochondrial damage; *etc.*) and verified the protective effect of antioxidants and estrogens (Michelucci et al., 2015; Paolini et al., 2015; Michelucci et al., 2017a; Guarnier et al., 2018).

Then the discovery of the sites of SOCE (i.e. CEUs) - in an apparently unrelated project - allowed us to revisit some structural modifications induced by ablation of Casq1 (presence of SR-stacks at the I band; Boncompagni et al., 2012) and understand how Casq1-null mice use continuously external Ca^2+^ to compensate for the reduced amount of Ca^2+^ in the SR stores (Michelucci et al., 2020).

In the end, it was quite an exciting journey that was worth traveling. What the future will bring is still unknown. But one idea is already in our mind: IVCT, the gold standard procedure to assess susceptibility to MH (test in which biopsies are exposed to increasing halothane of caffeine), requires the presence of extracellular Ca^2+^ to determine which patients are at risk of MH reactions (Fletcher et al., 1990). Hence, Ca^2+^ entry plays a role in contracture of biopsies and MH susceptibility. We have also recently showed that assembly of CEUs during exercise contributes to the increase in body temperature during exertional stress (Girolami et al., 2022). One puzzling question to answer in our future work would be: the constitutively active SOCE in Casq1-null fibers may contribute to the MH/HS susceptibility of these mice?

## Abbreviations

Casq, calsequestrin; CASQ, calsequestrin gene; CCD, Central Core Disease; CEU, Ca^2+^ entry unit; CPVT, catecholaminergic polymorphic ventricular tachycardia; CRU, Ca^2+^ release unit; DHPR, dihydropyridine receptor; EC coupling, excitation-contraction coupling; EDL, extensor digitorum longus; EM, electron microscopy; ER, endoplasmic reticulum; ES, exertional stress; FDB, flexor digitorum brevis; HS, heat stroke; IVCT, in vitro contracture test; mSOF, mitochondrial superoxide flashes; MHS, malignant hyperthermia susceptibility; NAC, N-acetylcysteine; PSS, porcine stress syndrome; ROS and NOS, oxidative species of oxygen and nitrogen; RyR, ryanodine receptor; SR, sarcoplasmic reticulum; SERCA, sarco-endoplasmic reticulum Ca^2+^ ATP-ase; SOCE, store-operated Ca^2+^ entry; TT, transverse tubule; WB, western blot; WT, wild type.
